# Inducing a summer brood break increases the efficacy of oxalic acid vaporization for *Varroa destructor* (Mesostigmata: Varroidae) control in *Apis mellifera* (Hymenoptera: Apidae) colonies

**DOI:** 10.1093/jisesa/iead085

**Published:** 2023-12-06

**Authors:** Jennifer A Berry, S Kris Braman, Keith S Delaplane, Lewis J Bartlett

**Affiliations:** Department of Entomology, University of Georgia, Athens, GA 30602, USA; Department of Entomology, University of Georgia, Athens, GA 30602, USA; Department of Entomology, University of Georgia, Athens, GA 30602, USA; Department of Entomology, University of Georgia, Athens, GA 30602, USA; Center for the Ecology of Infectious Diseases, Odum School of Ecology, University of Georgia, Athens, GA 30602, USA

**Keywords:** Varroa mite, beekeeping, IPM, oxalic acid, parasite control

## Abstract

The ectoparasitic mite, *Varroa destructor* (Anderson and Trueman), is the leading cause of western honey bee colony, *Apis mellifera* (L.), mortality in the United States. Due to mounting evidence of resistance to certain approved miticides, beekeepers are struggling to keep their colonies alive. To date, there are varied but limited approved options for *V. destructor* control. Vaporized oxalic acid (OA) has proven to be an effective treatment against the dispersal phase of *V. destructor* but has its limitations since the vapor cannot penetrate the protective wax cap of honey bee pupal cells where *V. destructor* reproduces. In the Southeastern United States, honey bee colonies often maintain brood throughout the year, limiting the usefulness of OA. Prior studies have shown that even repeated applications of OA while brood is present are ineffective at decreasing mite populations. In the summer of 2021, we studied whether incorporating a forced brood break while vaporizing with OA would be an effective treatment against *V. destructor*. Ninety experimental colonies were divided into 2 blocks, one with a brood break and the other with no brood break. Within the blocks, each colony was randomly assigned 1 of 3 treatments: no OA, 2 g OA, or 3 g OA. The combination of vaporizing with OA and a forced brood break increased mite mortality by 5× and reduced mite populations significantly. These results give beekeepers in mild climates an additional integrated pest management method for controlling *V. destructor* during the summer season.

## Introduction

*Varroa destructor*, an ectoparasitic mite, remains the leading biotic cause of western honey bee (*Apis mellifera* L.) mortality worldwide (Guzmán-Novoa et al. 2010, Rosenkranz et al. 2010, Kulhanek et al. 2017, Beyer et al. 2018). Controlling this pest has proven extremely difficult on several fronts (Bartlett 2022). First, *V. destructor* has developed resistance to several approved miticides (Elzen et al. 2000, Thompson et al. 2002, Rodríguez-Dehaibes et al. 2005, González-Cabrera et al. 2016, Rinkevich 2020) due to the limited number of chemical controls and their overuse. Second, *V. destructor*’s reproductive phase occurs inside the bee brood cell underneath a protective wax cap (Rosenkranz et al. 2010). Most miticides approved for use inside honey bee colonies do not penetrate this wax capping, rendering reproducing mites safe from treatment exposure (Rademacher and Harz 2006, Roth et al. 2020).

Chemical treatments are not the only route to mitigate the detrimental effects of *V. destructor*. Integrated pest management (IPM), a multipronged approach using biological, cultural, genetic, and chemical methods to control pests, has been successful in an array of agricultural and nonagricultural contexts (Frisbee and Luna 1989). The concept of IPM was introduced to the beekeeping community decades ago, but beekeeper adoption has remained slow. However, persistent mite resistance to chemical control agents and the mite’s continued impact on the industry have sustained IPM interest in some beekeeping sectors (Delaplane et al. 2005, Roth et al. 2020, Jack and Ellis 2021).

There are a few cultural options to limit *V. destructor* reproduction. One such option is brood cycle disruption, in which colonies are artificially forced to suspend reproduction and enter a period of broodlessness, forcing all *V. destructor* out of the protection of the brood cells (Gregorc et al. 2017, Al Toufailia et al. 2018, Jack et al. 2020). However, this and other cultural methods on their own have had limited success in reducing populations of *V. destructor* (Ellis et al. 2001, 2009, Delaplane et al. 2005, Harris 2007, Berry et al. 2010, 2012), meaning that beekeepers still mostly rely on chemical controls.

Organic acids, such as crystallized oxalic acid (OA) dihydrate, have been used for decades in Europe and Canada (Johnson et al. 2010) since they have proven extremely effective at killing *V. destructor* with little evidence for mite resistance (Adjlane et al. 2016, Maggi et al. 2016). Recently, OA was approved for use even while honey for human consumption is on colonies (EPA Reg. No. 91266-1), making it even more popular among beekeepers.

Trickling, vaporizing, and spraying OA are the 3 methods approved for use in honey bee colonies (Rademacher and Harz 2006). Trickling or spraying in which OA is suspended in a sugar water solution, is primarily used during winter months when there is no brood, and the bees are in a cluster (Charriére and Imdorf 2002, Rademacher and Harz 2006). Vaporizing OA is a method whereby one heats the crystals to 101 °C using a heating device (vaporizer) which forms a gas that permeates throughout the colony (Rademacher and Harz 2006). OA vaporization was originally intended for winter months, but it has increasingly been adopted by beekeepers as a warm-season treatment. None of these 3 treatments penetrates wax cappings and, by extension, affects only *V. destructor* adults on combs or adult bees (Adjlane et al. 2016, Ramsey et al. 2019). As such, they have proven ineffective against *V. destructor* during brood rearing seasons (Berry et al. 2022).

Several studies have tested the effort of a brood break and shown that it contributes to reducing mite levels (Wagnitz and Ellis 2010, Lodesani et al. 2014, Giacomelli et al. 2016, Gregorc et al. 2017). Additionally, studies looking at OA application during natural periods of broodlessness have highlighted that the lack of brood increases effectiveness (Al Toufailia et al. 2018). However, incorporating an induced brood break alongside OA vaporization application has been studied only once to date (Jack et al. 2020). The results were not favorable at the labeled 1 g per brood chamber of vaporized OA. Also, a number of colonies died due to the queen being caged for 24 days to induce a brood break late in the season.

The objective of this research was to determine if incorporating an earlier, shorter summer brood break (compared to Jack et al. 2020) along with higher application doses of 2 g or 3 g OA per brood chamber would improve the efficacy of summertime OA treatments in reducing *V destructor* parasitism rates.

## Materials and Methods

### Experimental Design

Experimental *A. mellifera* colonies were each established and maintained in two 8-frame-deep, Langstroth hives on research sites maintained by the research group. Only healthy queenright colonies, apart from high *V. destructor* parasite levels, were selected for the experiment. Each was headed by an open--mated queen.

In June 2021, 81 experimental colonies were placed across 7 research apiaries. All colonies had naturally occurring *V. destructor* infestations. No additional colonies were present in the apiaries. Each colony was assigned to 1 of 6 total treatments, whereby colonies were either subjected to a brood break or no brood break and dosed with either 0 g, 2 g, or 3 g of OA vapor per brood box. Colony assignments ensured that each treatment was represented in every apiary at least once.

### Brood Break

A brood break was induced by placing the queen onto an empty drawn frame in an 8-frame-deep super above a queen excluder. The remaining 7 frames of the super were completely honey bound, leaving no empty cells for the queen to lay eggs outside of her 1 designated frame. The queen remained in the deep super for 14 days. On day 14, the queen excluder was removed allowing the queen to roam freely. The frame she was given to lay eggs in was removed and destroyed. Additionally, frames in the other 2 supers that were isolated from the queen for 14 days were inspected for queen cells. None were detected. We chose 14 days for 2 reasons. One, for worker bees, the developmental time from egg to adult emergence is normally around 21 days, with brood being capped between days 8 and 9 (Winston 1987). Isolating the queen from the brood nest for 14 days, and then treating with OA on day 21, would ensure little to no capped brood within the brood nest along with *V. destructor* being in the dispersal stage (Rosenkranz et al. 2010). Second, we choose to isolate the queen for 14 days as opposed to 21–24 days to avoid colony issues much like what Jack et al. (2020) experienced in their study.

### Oxalic Acid Application

OA application was administered to colonies by vaporizing crystals (OAV) and the manufacturer’s instructions for the OxaVap ProVap 110 Vaporizer (OxaVap, Manning, SC). It is recommended to seal all openings before treating with OA to ensure the vapor will not leak out of the hive. We used blue shop towels to close the entrances, and screened bottom boards were sealed using corrugated plastic boards. A Champion 2000-watt gasoline generator was used to power the vaporizer and heat its chamber bowl to 230 °C. Depending on treatment group, 2 or 3 grams OA of solid dihydrate crystals per deep brood box were placed into the separated Teflon lid and then inserted into the chamber. The device was then turned right side up to force the OA crystals into the heated chamber, which generated the gaseous OA. Immediately, the device’s extended nozzle was inserted into each of the colony entrances and left for 30 s to ensure all of the OA crystals were vaporized and delivered into the colony. After the allotted time, the device was removed, but all materials used to seal the hive remained for an additional 10 min per colony. All personnel applying OA wore a full-face respirator equipped with OV/P100 cartridges.

### *Varroa destructor* Abundance and Mortality Measures

We quantified per-capita *V. destructor* parasitism levels, measured as “percent mite intensity” (varroa mites per 100 bees) for each colony using alcohol washes at 4 time points—Timepoint 1 on Day 0 prior to the induction of brood breaks, Timepoint 2 on Day 14 after the removal of queen excluders on brood break colonies, Timepoint 3 on Day 24 3 days after treatment with OA, and Timepoint 4 on Day 31 a month from the start of the experiment. Analysis focuses on a delta percent mite intensity (∆PMI) measure, which corresponds to the change in mites per 100 bees from Timepoint 1 or Timepoint 4.

For each alcohol wash for each colony at each timepoint, ~300 adult bees were collected from the brood nest (Dietemann et al. 2013) and placed into a Varroa EasyCheck device (Mann Lake, Hackensack, MN—manufactured by Véto-pharma) filled with 70% ethanol, which euthanizes adult bees and *V. destructor*. The lid to the Varroa EasyCheck was tightly sealed and vigorously shaken for 60 s. This action dislodges *V. destructor* from the adult bees. Next, the mesh basket filled with the adult bees was lifted out of the container, leaving dislodged *V. destructor* behind in the alcohol which were then counted and removed from the container. The mesh basket with the adult bees was placed back into the container, agitated vigorously again for 60 s, removed, and *V. destructor* counted and removed. This process was repeated until there were no more *V. destructor* dislodged for 2 consecutive washes. For each alcohol wash, all adult bees were counted by hand and recorded. This gave an exact count of adult bees per sample which is used to calculate the number of *V. destructor* per bee.

To measure *V. destructor* mortality rates, Dadant (Hamilton, IL) wood-bound mite sticky boards (SB1) were inserted onto the floor of each colony for 72 h prior to OA vaporization to determine mite fall (Day 18–Day 21). On the day of OA vaporization (Day 21), the initial sticky boards were removed and new boards (SB2) inserted for an additional 72 h period (Day 21 to Day 24) including during the application of the OA for treated colonies. The total number of mites was counted on each sticky board as a measure of *V. destructor* mite fall immediately prior to and during the OA treatment period. Fold change in mite fall was calculated for each colony as the number of mites on sticky board 2 minus the number of mites on sticky board 1, divided by the number of mites on sticky board 1.

### Statistical Analyses

All analyses were undertaken using the statistical programming language R v.3.6.1. We used a generalized linear mixed-modeling framework paired with type-III ANOVAs via the “afex” package (Singmann et al. 2020) which wraps the “lme4” package (Bates et al. 2015) and compared effect sizes extracted from fit models using the “emmeans” package (Lenth 2020). We compared pretreatment and during/posttreatment mite mortality rates using linear mixed models with fold change in mite mortality as a response variable, brood break, and OA treatment as interacting fixed predictors, and apiary as a random effect. We examined per-capita mite parasitism using generalized mixed models with a Poisson error structure, with timepoint (continuous), brood break, and OA as interacting fixed predictors, and nested random effects of colony and apiary to account for the time series analysis and geographic grouping of the replicates. All data and analysis for this study are made openly and freely available via a GitHub repository archived on Zenodo (DOI: 10.5281/zenodo.8388042).

## Results

### *Varroa destructor* Mortality

**Fig. 1.**
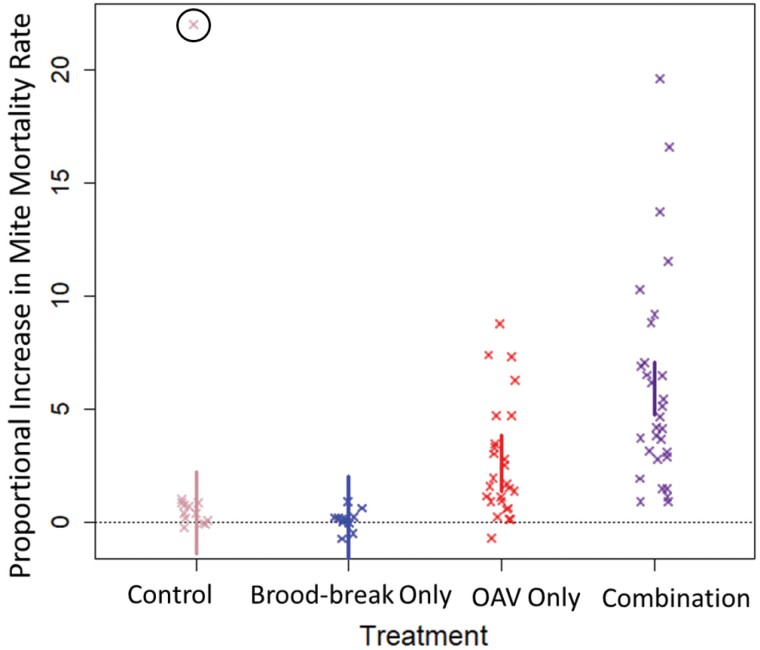
Comparison of change in mite mortality rate by treatment group (control, brood break only, oxalic acid vaporization [OAV] only, and a brood break combined with OAV), calculated using mite drop 72 h prior to treatment and 72 h during and posttreatment. Both the OAV treatments and combination treatment saw significant increases in inferred mite mortality, with the combination treatment significantly higher in this measure than the OAV treatment only. Each point represents a single colony. Vertical bars represent 95% confidence intervals around the mean. A “0” value corresponds to no change in mite mortality, a “1” value corresponds to a doubling of mite mortality, and a “5” value correspond to a 6-fold increase in mite mortality.

We found no evidence that the 3 g dose of OA killed more *V. destructor* than the 2 g dose (*t*_68.4_ = 0.54, *P* = 0.854). All onward analysis therefore treated OAV as a binary predictor; colonies were either treated or not treated with OA. We found strong evidence of a multiplicative interaction effect between inducing a brood break and treating with OA on our *V. destructor* mortality measure (*F*_71.03_ = 7.34, *P* = 0.008), shown in Fig. 1 where the combination treatment led to *V. destructor* mortality rates 6× higher (95CI 3.9× − 8.2×) than background while treated colonies without a brood break saw 72 h mortality rates rise only 2.9× higher than background (95CI 0.70× − 5.0×). As expected, colonies which were not treated with OA showed no significant change in mite mortality rates during the treatment window, whether they experienced a brood break (0.457×, 95CI −2.2× − 3.1×) or not (2.2×, 95CI −0.37× − 4.7×), see Fig. 1. Simply stated, inducing a brood break more than doubled the effectiveness of OA treatment on our acute *V. destructor* mortality measure.

### Percent Mite Intensity

**Fig. 2.**
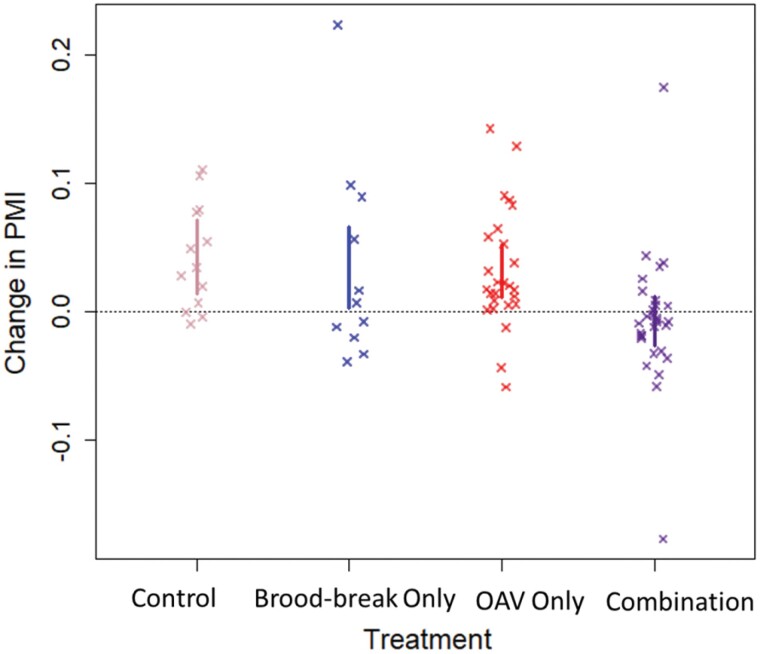
Summary of the change in percent mite intensity (“PMI” or mites per 100 bees) by treatment (control, brood break only, oxalic acid vaporization [OAV] only, and a brood break combined with OAV), calculated using mite washes prior to and after treatment periods. Colonies experiencing a brood break alongside an OAV treatment had a significant decrease in mite infestation. Colonies with no brood break, brood break or OAV treatment showed a significant increase in mite parasitism rates of adult bees. Vertical bars represent 95% confidence intervals.

Concordant with the *V. destructor* mortality results, we found strong evidence of a multiplicative interaction effect on colony *V. destructor* percent mite intensity between inducing a brood break and OA treatment (χ^2^ = 5.65, *P* = 0.02), where *V. destructor* loads at the final timepoints measure were lowest in colonies experiencing the combined treatment (Fig. 2). Both an induced brood break (∆PMI = 0.15; 95CI 0.03–0.26) and oxalic treatment (∆PMI = 0.18; 95CI 0.11–0.24) alone showed increasing rates of mite parasitism with time over the course of the 31-day experiment; however, this was lower than those for the untreated control colonies (∆PMI = 0.22; 95CI 0.13–0.31). Only in the combined treatment did per-capita mite parasitism rates decrease over the course of the experiment (∆PMI = -0.11; 95CI −0.19 − −0.04), see Fig. 2. Across the 81 colonies in the experiment, the median PMI at the start of the experiment was 2.0%; this is the treatment threshold at which control is recommended (Honey Bee Health Coalition 2022). At the end of the experiment 31 days later, median PMI for control colonies was 5.0%, whereas for colonies treated with both OAV and a brood break, median PMI 1.8%, back below the treatment threshold.

## Discussion

We found that combining an induced brood break with oxalic vaporization at either 2 g or 3 g per brood box was effective at reducing *V. destructor* parasitism rates in colonies (Fig. 2). Treating with only a brood break or OA was insufficient at preventing parasitism rates from increasing during the summer. This is explained by the doubling of efficacy of OA vapor at killing *V. destructor* when used in conjunction with a forced brood break (Fig. 1), illustrating and substantiating the IPM tenet that different control approaches act multiplicatively to optimize pest control outcomes.

Induction of a brood break in summer allows for expanded effectiveness of OA use in beekeeping regions where a natural brood break may not occur. Other studies that have examined the effect of repeated applications of OA, either by liquid trickling or vaporization, have shown OA to be ineffective during the brood rearing season (Gregorc et al. 2017, Jack et al. 2020, 2021, Berry et al. 2022). While our work demonstrates a partial solution to this problem, we did so by using an oxalic dose currently above label rates. We justified this on the basis that another study in the same region also combined a brood break late in the season with OA (Jack et al. 2020) but showed limited effectiveness in reducing *V. destructor* using the legal dose of 1 g per brood chamber. In another study, the same authors (Jack et al. 2021) increased their dose of OA to 2 g and 4 g while brood was present, significantly lowering levels of *V. destructor* compared with those only vaporized with 1 g per brood chamber. Al Toufailia et al. (2018) found similar results of increased mortality while vaporizing twice with 2.25 g of OA during the broodless winter months. Due to the results of these studies, we decided to incorporate doubling and tripling the amount of OA to be vaporized alongside a brood break.

Owing to the high colony mortality Jack et al. (2020) experienced due to the queen being caged for 24 days, we chose not to cage the queen but instead restrict her into a third super above a queen excluder with full access to only 1 drawn frame. The other frames in the third super were completely covered in capped honey, which we visually confirmed. By allowing the queen to lay, even though in a restricted area, the colony effectively is queenright which prevents the construction of queen cells. In addition, this approach does not require to find the queen; colonies can be shaken into the excluded super and the queen assumed segregated from the lower 2 brood chambers. This increases the viability of the technique amongst commercial beekeepers for whom labor costs of finding and caging queens may be prohibitive.

There is a critical need for additional IPM options for controlling *V. destructor* during the brood rearing months. Resistance to some and limiting factors for other miticides has left beekeepers with few options for controlling *V. destructor* (Elzen et al. 2000, Thompson et al. 2002, Rodríguez-Dehaibes et al. 2005, González-Cabrera et al. 2016, Rinkevich 2020). OA is becoming the chemical treatment of choice for beekeepers for many reasons already stated (Jack and Ellis 2021, Bartlett 2022). Based on our results, we recommend that beekeepers employ OA vaporization during a brood break, either naturally occurring or induced. Implementing a brood break into a beekeeper’s treatment regime can be easily accomplished and combined with an OA treatment to reduce *V. destructor* populations during critical summer months when few other control options are available. There are other periods during the beekeeping calendar when beekeepers can take advantage of a brood break and vaporize with OA, for instance, when a colony has swarmed or when beekeepers are making splits or requeening. Anytime there is no capped brood, that is, mites are exclusively in the dispersal phase (Rosenkranz et al. 2010, Nazzi and LeConte 2016), there is an opportunity for this treatment method.

Increasing the dose of OA has also been shown to be beneficial in increasing *V. destructor* mortality in ours and other studies (Jack et al. 2021), but to date, the legal amount for use in honey bee colonies is only 1 g per brood chamber (US EPA. 2015). We advocate efforts to modify the OA label to approve use at higher doses.
